# The influence of gamification on the teaching assessment of human anatomy

**DOI:** 10.3205/zma001860

**Published:** 2026-06-15

**Authors:** Alberto García-Barrios, Ana Isabel Cisneros-Gimeno, Jesús Benito-Rodríguez, Jaime Whyte-Orozco, Encarcacíón Rubio-Aranda

**Affiliations:** 1 University of Zaragoza, School of Medicine, Department of Human Anatomy and Histology, Zaragoza, Spain; 2 Health Research Institute of Aragón, Medical and Genetic Research Group (GIIS099), Aragón, Spain; 3 Antecessor B51_23D (Gobierno de Aragón), Aragón, Spain; 4 University of Zaragoza, School of Medicine, Department of Microbiology, Paediatrics, Radiology and Public Health, Zaragoza, Spain; 5 B43_23R: Agua y Salud Ambiental (Gobierno de Aragón), Aragón, Spain

**Keywords:** anatomy, medical education, gamification, health professions education

## Abstract

Anatomy teaching in medical schools has experienced a reduction in teaching hours in recent decades, which has led to the implementation of innovative methodologies, such as gamification and serious games, to improve student learning and motivation. In this study we evaluated the impact of these tools on the final grades of students enrolled in human anatomy I: Locomotor system for four consecutive academic years (2020-2024).

Two groups were compared: one with traditional methodology and another that integrated gamification activities, and in which the content, teaching staff and assessment criteria remained unchanged.

Students who participated in gamified teaching obtained significantly (p<0.005) better grades than the control group in the four teaching courses studied. However, no significant differences were observed between sexes or between the different academic years.

Gamification in higher education, in addition to motivating and energising classes, is an effective and versatile tool for optimising academic performance in the health sciences.

## Abstract (de)

In den letzten Jahrzehnten ist die Zahl der Unterrichtsstunden im Anatomieunterricht an medizinischen Fakultäten zurückgegangen, was zur Einführung innovativer Methoden wie Gamification und Serious Games geführt hat, um die Lernleistung und Motivation der Studierenden zu verbessern. In dieser Studie wurde der Einfluss dieser Instrumente auf die Abschlussnoten der Studierenden im Fach Humananatomie I: Bewegungsapparat über vier aufeinanderfolgende Studienjahre (2020–2024) untersucht.

Es wurden zwei Gruppen verglichen: eine mit traditioneller Methodik und eine, in die Gamification-Aktivitäten integriert wurden, wobei Inhalte, Lehrpersonal und Bewertungskriterien unverändert blieben.

Studierende, die an gamifizierten Lehrveranstaltungen teilnahmen, erzielten in den vier untersuchten Studienjahren signifikant bessere Noten als die Kontrollgruppe (p<0,005). Signifikante Unterschiede zwischen den Geschlechtern oder den einzelnen Studienjahren zeigten sich jedoch nicht.

Gamification in der Hochschulbildung ist neben ihrer motivierenden und aktivierenden Funktion im Unterricht ein effektives Instrument zur Verbesserung der Studienleistungen in den Gesundheitswissenschaften.

## 1. Introduction

The amount of time devoted to the study of anatomical sciences in medical schools varies around the world, as it is necessary to have a good foundation for the professional development of future physicians, regardless of their specialty [1], and to establish a relationship between anatomical knowledge and clinical experience to enable students to develop diagnostic reasoning skills [2]. However, despite its importance, the vast majority of universities have reduced the number of hours devoted to the study of anatomy in recent decades [3], [4], [5]. 

The reduction of time allocated to anatomy teaching and the presence of new generations, Generation Z or Digital Natives, in higher education classrooms have stimulated the emergence of new and innovative teaching methodologies (problem-based learning, 3D platforms, gamification and game-based learning) to address the teaching and learning process of students, and to improve the confidence of new graduates in their anatomical knowledge [6], [7].

Gamification, in particular, is a novel alternative that involves the use of game-like features in non-game contexts and teaching-learning sessions for the purpose of acquiring knowledge [8], [9], [10], [11], [12], [13]. For its part, game-based learning involves the use of complete games designed specifically to facilitate the learning of content or skills, while serious games are developed with an explicit educational purpose that goes beyond entertainment [14].

Although game-based learning (GBL), serious games and gamification are usually associated with the same group, they are concepts with different characteristics [3], [15], [16]. Gamification uses game concepts to make the teaching and learning process more enjoyable and fun, GBL involves modifying a game to achieve a certain learning outcome, and serious games are created specifically for teaching and learning purposes [8], [11], [15], [17], [18], [19].

Several studies have shown that these strategies can improve student engagement, promote meaningful learning, and strengthen key skills such as clinical reasoning, decision-making, teamwork, and problem-solving in safe and controlled environments [20], [21], [22], [23]. In medical education, serious games and gamification have been successfully applied in areas such as anatomy teaching, clinical simulation, surgical skills training, interprofessional education, and formative assessment [24], [25].

The use of gamification is widely implemented in medical education, through the application of numerous new tools, but in most cases, studies approach gamification from a qualitative point of view based on student perception in relation to aspects such as student motivation, interaction or participation [26], [27].

The aim of this work was to evaluate the effect of the use of gaming, in all its variants (gamification, GBL and serious games) on the academic results of students in 4 consecutive teaching years (from 2020-2021 to 2023-2024) in the subject of human anatomy I: Locomotor system.

## 2. Material and methods

### 2.1. Design of the study

A randomised controlled design was performed on a sample of 687 students enrolled in the subject “Human Anatomy I: Locomotor System” at the Faculty of Medicine of the University of Zaragoza, in four consecutive academic years (2020-2021, 2021-2022, 2022-2023 and 2023-2024). 

**Figure 1:**
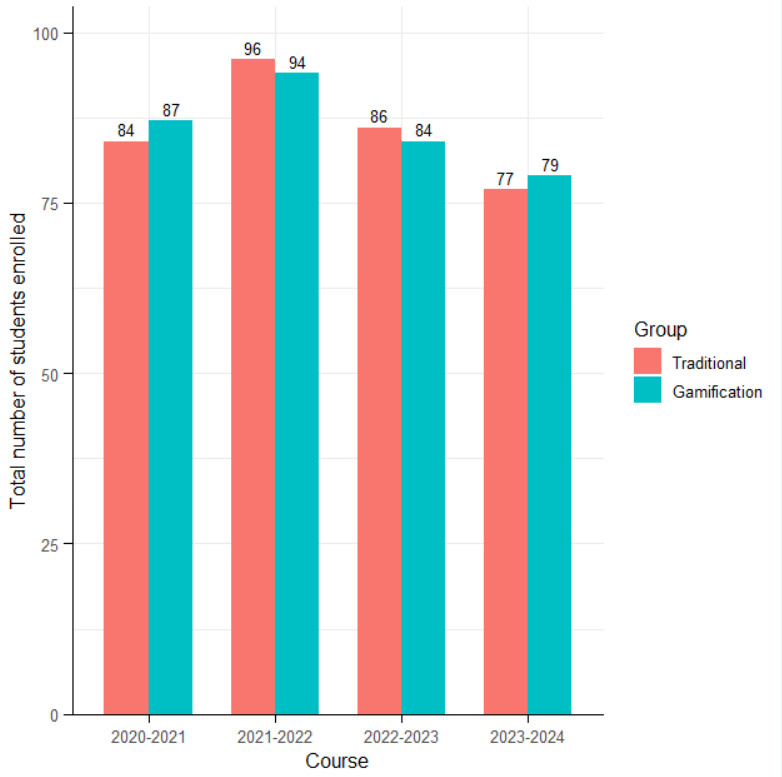
Distribution of the students included in the current study, divided into group traditional and group gamification

Students are randomly assigned at the time of enrolment through the Academic Management System based on the Sigma^®^ platform of the University of Zaragoza, in each of the two groups (group traditional and group gamification) established for the development of the degree in Medicine. Figure 1 (Fig. 1) shows the distribution of students enrolled by academic year and group.

This subject is taught in the second semester of the first year of the degree and is equivalent to 6 credits of the European Credit Transfer and Accumulation System (ECTS), and is didactically divided into 4 modules: Trunk, upper extremity, lower extremity and head.

The teaching methodology, according to the student guide, is based on theoretical lectures combined with the development of practical sessions carried out in the dissection room, where the study of the locomotor apparatus is carried out by means of prosection and dissection techniques of cadavers, description and visualisation of bone remains and different anatomical models. During the semester under study, students receive a total of 39 theory classes and 26 hours of practical classes (3 theory classes and 2 hours of practical classes per week), the latter of which are compulsory. 

During the four teaching courses, the students belonging to group traditional only used a teaching-learning methodology based on lectures and the development of traditional practices: recognition of anatomical structures in cadavers, prosections, models and bone remains from ossuaries. In group gamification, a teaching methodology was implemented where, in addition to lectures and traditional practical teaching, a total of 4 activities to reinforce the content, were implemented in the practical sessions (lasting one hour each at the end of each module), which included the use of games in all their variants: gamification, GBL and serious games. In the four courses analysed, the teaching staff and curricular content remained unchanged, guaranteeing an identical educational experience. The final assessment of the subject, following the criteria set out in the teaching guide, was the same in both groups. 

### 2.2. Planned game tools

The different activities and game tools established, aimed at improving the skills and competences acquired during the development of each of the modules. 

#### 2.2.1. Module 1: Trunk – gamification

In the first module, the Kahoot^®^ platform was used as a gamification tool, where the design of a questionnaire was proposed, containing questions corresponding to the module, with 15 multiple choice questions with only one correct answer and a time limit of 20 seconds. Once the time for each question had elapsed, the result of the question was visualised and discussed in real time. 

The questions in this questionnaire were designed by the teachers responsible for the subject AGB and JBR and reviewed by the teachers AICG and JWO. 

For the use of Kahoot^®^, students were provided with instructions on how to access the activity and carry it out online or by downloading the application on mobile devices. 

#### 2.2.2. Modules 2 and 3: Upper and lower extremities – serious games 

For these two modules, the methodology proposed was to introduce two ‘serious games’ activities based on a breakout or virtual escape room, where the students had to manage to “escape” from the virtual rooms that had been created for this purpose. On the one hand, in module 2 they had to escape from the mythical temple of Angkor in Indonesia, while in module 3 they had to escape from the University Hospital. To achieve the objective, they had to solve a series of challenges and riddles, in a linear and sequential way, whose common thread was related to the theme corresponding to each of the modules. In total, 4 exercises were proposed for each activity based on the recognition of anatomical structures in models, prosection and cadaver pieces and clinical cases associated with the content of each of the modules. 

In order to generate the activity, which was carried out entirely on electronic devices, the teacher AGB was in charge of developing the content, which was subsequently reviewed by JBR, AICG and JWO, through the online platform Genially^®^. At the time of the activity, the students were provided with the necessary link to access it. 

#### 2.2.3. Module 4: Head – game-based learning

In the last module, a series of interactive images were created using the online platform Educaplay^®^, in which the students had to recognise and mark a series of anatomical structures, based on their own images taken in the dissection sessions, related to this module, as they were asked to do so by the platform. In total, they had to recognise a total of 30 anatomical structures. 

The images were prepared and reviewed by the teaching staff responsible (AGB, JBR, AICG and JWO).

### 2.3. Statistical analysis

#### 2.3.1. Statistical methodology

The study population consisted of the students enrolled (687 students) in the subject of Human Anatomy at the Faculty of Medicine of Zaragoza during four consecutive academic years: 2020-21, 2021-22, 2022-23 and 2023-24. The statistical study was carried out by ERA.

Variables to be studied: The main variable to be studied was the numerical grade obtained in the final exam of the subject: as explanatory variables were used, the type of teaching methodology received: gamification, master class, gender and academic year.

#### 2.3.2. Statistical analysis

Quantitative variables were described using the mean and standard deviation, and categorical variables were described using frequency and percentages.

Given the size of the groups and following the central limit theorem, the t-Student statistic and the 95% confidence interval were used to compare the marks obtained according to the teaching methodology received in the four years analysed. The comparison of the marks obtained over the four years for each of the two teaching methodologies used was carried out using the one-factor ANOVA test.

## 3. Results

Human anatomy I: Locomotor system is a compulsory subject taken during the second semester of the first year of the bachelor’s degree in medicine at the University of Zaragoza. In this subject, as in the rest of the subjects of the degree and other degrees in health sciences at the University of Zaragoza, there is a predominance of female students over male students in all the academic years studied, with the female:male ratio being (2.8:1) in the academic year 2020-2021; (3.5:1) in the academic year 2021-2022; (2.5:1) in the academic year 2022-2023 and (3.8:1) in the academic year 2023-2024. On the other hand, the number of students enrolled for the second time or more in this subject was slightly higher, although not significant, in group 1 than in group 2 (3% vs. 1.5%). 

**Figure 2:**
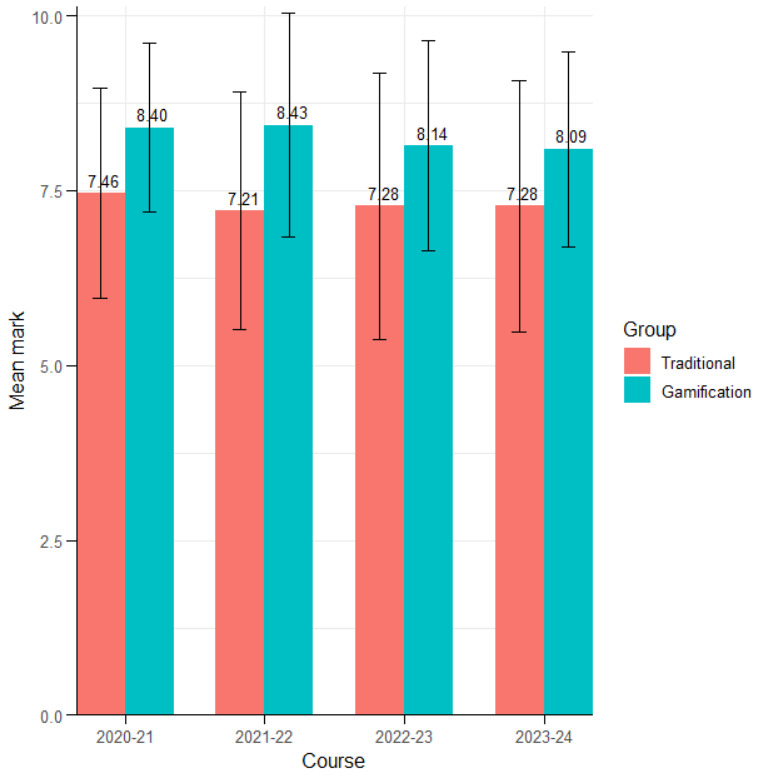
Results of the final evaluation per teaching year in group traditional teaching and group traditional teaching and gamification

The mean scores and their standard deviations obtained in both groups across the four academic years analyzed are shown in figure 2 (Fig. 2).

**Figure 3:**
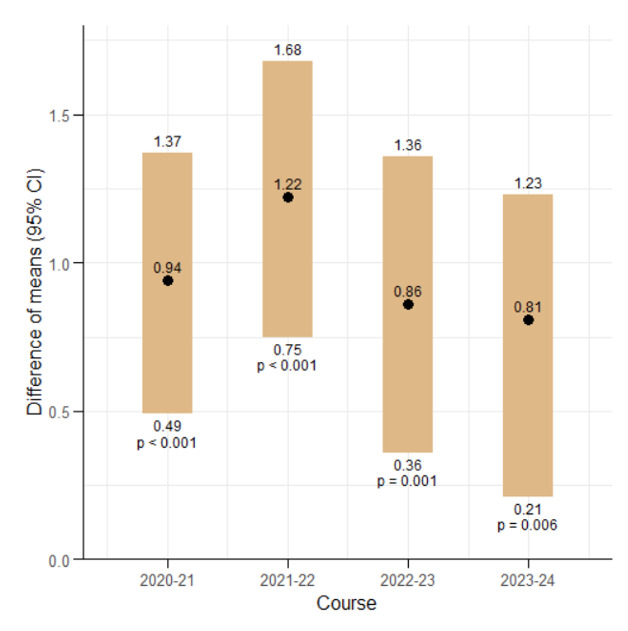
Estimation of the difference in grades between two learning methods (95% CI) across the four academic years analyzed

In the four courses analysed (see figure 3 (Fig. 3)), statistically significant differences can be seen in the marks obtained in the final assessment of the subject according to the type of teaching received (“traditional teaching” vs. “traditional teaching and gamification”, the latter being better when traditional teaching is complemented by gamification activities. 

The greatest difference was found in the 2021-22 academic year, with a 95% confidence of between 0.75 and 1.68 points more for students with gamified teaching, and the smallest in the 2023-24 academic year, in which students with gamified teaching obtained between 0.21 and 1.23 points more than their classmates who received traditional teaching.

When comparing the marks obtained by students who had received traditional teaching during the years analysed, no significant differences were observed. The same was true when comparing the grades obtained by students who had received gamified instruction. Nor were significant differences observed when comparing the marks obtained by females and males in any of the four teaching courses.

## 4. Discussion

The evolution in the teaching of anatomy at the beginning of the 21^th^ century has brought about significant changes in the implementation of medical curricula, driven by the entry into force of new medical subjects and by the change in pedagogical approaches, which have generally led to a reduction in teaching hours in subjects such as anatomy. On the other hand, the change in student profiles and the different learning styles [28], [29], reflect the need to generate a motivating classroom environment and more dynamic learning through more innovative teaching methodologies, such as gamification in its various versions. Likewise, and as stated by authors such as Anyanwu and Blakeli et al. [28], [30], we consider that the creation of a more dynamic learning environment through different learning strategies is necessary to improve student attention and participation.

In the practical sessions, the students are more participative than in the theoretical lecture sessions, as the atmosphere is more favourable and the group is smaller. However, a certain “passivity” and lack of interactivity with the group is still observed [31], [32], [33]; therefore, with these strategies we get each student to be the epicentre of his/her own teaching-learning process by actively participating in it, improving participation, attention, satisfaction and motivation in an individualised way and reducing the concern for learning the subject [29], [34], [35], [36]. 

The use of gamification in higher education is proposed as a tool to break the monotony that can be established in theoretical-practical sessions by improving the classroom climate and fostering a more positive learning environment. In fact, as proposed by other authors [1], [19], [37], and even by our team in previous studies [30], [31], [32], the use of new teaching methodologies, including gamification, game-based learning and serious games, are qualitatively highly valued by students for their positive effect on motivation, participation, integration of theoretical and practical content, group cohesion and improvement in the classroom climate, factors which, as we have observed in our study, are of significant importance for obtaining better grades in the final assessment. Likewise, and as we have seen in previous studies [38], [39], tools based on serious games are more highly valued by students for helping to integrate theoretical and practical content, as well as group cohesion, as it is a sequential activity that encompasses a greater amount of content in the different phases of its development.

In this case, we have proposed the use of gamification tools, only in one group (group 2), to compare the qualitative effect, through subjective evaluation surveys conducted in each of the courses in which the study was carried out, where students assess the positive effect these activities have on their learning and on improving the classroom environment by fostering relationships between students, and between students and teachers, on the students and the classroom climate, but also the quantitative effect when facing the final assessment, on the other group that we use as a control group (group 1). Despite carrying out these activities during the hours allocated to practical sessions, accounting for 14% (4 hours out of 26) of the total time, this does not detract from the experimental group, as the content taught in each of the modules is used in these activities as a review.

Although there are different studies that have assessed the positive effect of the use of games in the quantitative assessment of students, these have focused only on the use of a specific gamification alternative. Thus, we find authors who have used gamified questionnaires on platforms such as Kahoot^®^ [40], GDRS Sidra [41] or self-made [1], [42]; others who have proposed game-based learning (GBL) activities [19] or through serious games [18], [43], and even activities based on images [44], but none of them together in a single study as we have done in our case, where we have been able to demonstrate the positive effect of gamification, regardless of the way it is implemented in the classroom, on the quantitative assessment of students.

One of the strong points of our study is that all the activities centred on the use of games were carried out in the practical sessions of the subject, which were compulsory and taught by the same teachers, and therefore carried out by all the students. Likewise, participation in the development of these activities did not generate an extra mark on the final mark for the subject, so as not to discriminate against the control group that did not carry them out. However, in order to generate extrinsic motivation in those students who participated in the experience and achieved better results in these activities, they received a bonus of participation in an anatomical dissection workshop, once the teaching and assessment period for the subject was over. This fact corroborates the positive effect of the use of gamification in the quantitative assessment of the subject. 

On the other hand, and although it could be considered a limitation to compare a control group and one with gamification, and that there could be previous differences in the students' ability, this has been carried out for 4 consecutive years, always obtaining better grades in the group where gamification has been taught compared to the control group. In addition, students entering anatomy studies tend to be very homogeneous, as they require a high cut-off mark and uniform study skills in order to access the degree.

As a future line of research that could corroborate the improvement in long-term learning, it might be worth comparing the grades of the same study groups in the OSCEs (Objective Structured Clinical Evaluations) carried out on undergraduate medical students at the University of Zaragoza before they complete their studies.

## 5. Conclusion

The introduction of gaming, regardless of modality, in the higher education classroom brings about a quantitative improvement in the student learning process, evidencing a higher rating compared to the sole use of more “traditional” teaching.

## Authors’ ORCIDs

- Alberto García-Barrios: [0000-0001-5560-3771]
- Ana Isabel Cisneros-Gimeno: [0000-0002-5494-343X]
- Jaime Whyte-Orozco: [0000-0001-9372-4267]
- Encarnación Rubio-Aranda: [0000-0002-9273-5885]

## Competing interests

The authors declare that they have no competing interests.
